# Intravenous ondansetron reduced nausea but not pruritus following intrathecal morphine in children: Interim results of a randomized, double‐blinded, placebo‐control trial

**DOI:** 10.1111/pan.14517

**Published:** 2022-07-12

**Authors:** Elizabeth M. Putnam, Rebecca A. Hong, John M. Park, Ying Li, Aleda Leis, Shobha Malviya

**Affiliations:** ^1^ Department of Anesthesiology University of Michigan Health System Ann Arbor MI USA; ^2^ Department of Urology University of Michigan Health System Ann Arbor MI USA; ^3^ Depatment of Orthopaedic Surgery University of Michigan Health System Ann Arbor MI USA; ^4^ Department of Epidemiology University of Michigan Health System Ann Arbor MI USA

**Keywords:** intrathecal morphine, ondansetron, pediatrics, postoperative nausea, pruritus, randomized controlled trial

## Abstract

**Study Objective:**

This study's purpose was to determine if ondansetron can prevent pruritus after administration of intrathecal morphine in children, as has been demonstrated in adults.

**Design:**

A double‐blinded, randomized placebo‐controlled trial.

**Setting:**

Operating room and first 24 h postoperative inpatient stay at an academic children's hospital.

**Patients:**

Forty‐six children aged 3–17 years, who received 4–5 mcg/kg intrathecal morphine for urological or orthopedic procedures were included.

**Interventions:**

Children were randomized to receive intravenous ondansetron (treatment) or saline placebo (placebo), prior to intrathecal morphine administration, and q6H for 24 h thereafter. Intraoperative anti‐emetics and postoperative rescue treatments for pruritus and nausea were standardized.

**Measurements:**

Patients were interviewed q6H for scored pruritus, nausea, and pain, using standardized scales.

**Main Results:**

The trial was terminated for futility after interim analysis. Forty‐six children were recruited and 45 completed data collection. No significant difference was found between both groups for incidence of pruritus (requiring treatment) [relative risk (RR) 0.9, 95% CI: 0.7, 1.2], during the first postoperative 24 h. Notably, the incidence of pruritus was 84% overall, much higher than rates in previously published studies. Intravenous ondansetron significantly reduced the incidence of nausea, compared with the placebo group [RR 0.5, 95% CI: 0.3, 0.9].

**Conclusions:**

This study found no evidence for intravenous ondansetron as an effective preventative for pruritus following intrathecal morphine in children. However, this RCT did find that the rate of pruritus following intrathecal morphine administration may be significantly higher than previously thought. Nausea and vomiting (a secondary outcome) were reduced significantly in the treatment group. The negative findings of this study reinforce the potential dangers of extrapolating the drug effects seen in adults onto pediatric patients.

## 1 INTRODUCTION

Pruritus is one of the most common and bothersome side effects of intrathecal morphine in children, with a reported incidence of 30%–60%.^1^, ^2^, ^3^ In a retrospective review of intrathecal morphine for urologic surgery in children, a study from our institution found a 40% incidence of pruritus.^2^ Clinicians who have witnessed unbearable itching and scratching in their young patients after intrathecal morphine may be reluctant to offer this effective pain control to future patients for fear of these unpleasant sequelae.

5‐HT_3_ antagonists are considered among the first line of treatments for both morphine‐induced nausea and pruritus in adult, particularly obstetric, populations.^4^, ^5^, ^6^, ^7^, ^8^ A systematic review of randomized controlled trials (RCTs) in adults, found that prophylactic 5‐HT_3_ antagonists were effective when compared to placebo, in reducing pruritus from neuraxial opioids.^9^ However, this review found evidence of publication bias. Other studies have demonstrated that both preemptive and around‐the‐clock dosing of ondansetron reduces pruritus (both incidence and intensity) in adults who received intrathecal opioids.^10^, ^11^

Case reports suggest ondansetron may be an effective treatment for pruritus in children,^12^, ^13^, ^14^ but to date there are no data to support the efficacy of 5‐HT_3_ antagonists in preventing or treating intrathecal morphine‐related pruritus in this age group. In contrast, antihistamines such as diphenhydramine, while used in many pediatric settings, have been shown to be ineffective for treating opioid‐related pruritus.^1^, ^3^ This is corroborated by research in primates.^15^

The mechanism of morphine‐induced itch is not fully understood.^1^, ^8^, ^16^, ^17^ Neuraxial opioids induce more frequent and intense itch than peripherally administered doses.^18^ Centrally located μ‐opioid receptors, in the medullary dorsal horn, may play a role. Activation of this site, by opioid injection, induces a dose‐related scratch frequency in primates.^15^ However, the physiology is more complex, as morphine‐induced pruritus is not always reversed with opioid receptor antagonists, such as naloxone.^4^ Effective treatments for neuraxial opioid induced pruritus in humans include partial opioid agonists,^1^, ^4^, ^19^ and nonopioid receptor agents, including 5‐HT_3_ antagonists,^5^, ^6^, ^20^, ^21^ propofol,^4^, ^22^ prostaglandin modulation,^8^ and low‐dose continuous naloxone infusions.^23^

We therefore undertook the following prospective randomized double‐blinded placebo‐controlled trial: The primary objective was evaluation of efficacy of ondansetron as an anti‐pruritic in children who received intrathecal morphine for orthopedic or urologic surgery. We hypothesized that preemptive and continued blocking of 5‐HT_3_ receptors with the 5‐HT_3_ receptor antagonist ondansetron, given intravenously, prior to intrathecal morphine, and every 6 h thereafter, would reduce the incidence and intensity of pruritus as a side effect of intrathecal morphine, in our pediatric patients. Our secondary outcomes were incidence of nausea, adequacy of pain relief and side effects of anti‐pruritic and anti‐emetic treatments.

## 2 MATERIALS AND METHODS

This RCT was registered prior to patient recruitment at ClinicalTrials.gov (NCT03262038; Principal Investigator: Elizabeth Putnam; date of registration: August 25, 2017). Approval was obtained from our institutional review board (HUM00124202) and the protocol was additionally approved by our departmental research committee. All parents/legal guardians gave written informed consent; assent was obtained from those children ≥10 years of age. The study was determined to be exempt from Investigational New Drug regulations from the Food and Drug Administration for off‐label use of ondansetron. This manuscript adheres to the applicable CONSORT guidelines.

Children were eligible for study inclusion if aged 3–17 years, ≤100 kg in weight, able to use a verbal or pictorial pain assessment tool and scheduled to receive intrathecal morphine for a urologic or orthopedic surgical procedure, typically including (but not limited to) pyeloplasty, ureteral re‐implantation and femoral osteotomy; intrathecal morphine being the standard of care for this surgical population at our institution. Patients undergoing posterior spinal fusions were excluded, as these patients received higher doses of intrathecal morphine.

Patients were excluded from this study if they were unable to use verbal or pictorial scoring scales, were hypersensitive to selective 5‐HT_3_ receptor antagonists, had a history of hypersensitivity to any of the anti‐emetics or anti‐pruritics included in the protocol, were using 5‐HT_3_ receptor antagonist medications regularly, were taking selective serotonin reuptake inhibitors (SSRIs) regularly, had a diagnosis of congenital long QT syndrome or severe hepatic impairment, were pregnant, or were nursing mothers.

Following parental consent, and patient assent where appropriate, patients were serially enrolled at the surgical clinic, or on the day of surgery. After obtaining consent, children were randomized in accordance with a computer‐generated schema that stratified by sex, 1:1 to either the ondansetron or placebo group. The schema was accessible only to the pharmacist preparing the drug/placebo for the patient. All patients, family members or caregivers, the medical team involved with patient care, and trained research assistants were blinded to study treatment group allocation.

### 2.1 Study treatments

The treatment group received ondansetron 0.1mg/kg, administered as an intravenous 5ml bolus, administered at least 15 minutes prior to the intrathecal morphine injection. This was followed postoperatively by identical ondansetron doses, every 6 h (q6H) for 24 h (not to exceed a maximum of 64mg in 24 h or 16 mg/dose, the maximum dose recommended by the FDA). The placebo group received an intravenous 5ml bolus dose of saline and around‐the‐clock placebo doses for 24 h after surgery, administered in an identical fashion. Both drugs were prepared by pharmacy in identical 5ml syringes, to ensure the blinding of all clinicians and subjects.

### 2.2 Intraoperative study protocol

The study took place at a single pediatric university hospital. The induction and maintenance of general anesthesia and pain medications were at the anesthesiologist's discretion. Intrathecal morphine dose was 4–5 mcg/kg. All intrathecal injections were confirmed by aspiration of CSF at the start and end of the intrathecal morphine injection. Intraoperative anti‐emetics were given according to the study protocol (see Appendix A): dexamethasone 0.15 mg/kg (max 4 mg) and diphenhydramine 0.3 mg/kg (max 12.5 mg), delivering the standard of care using departmental anti‐emetic administration protocols. Patients deemed at high risk for postoperative nausea and vomiting (PONV) according to our institution's standard of care, were given aprepitant (2–3 mg/kg up to 80 mg), a fluid bolus of at least 30 ml/kg, and total intravenous anesthesia (TIVA), or a sub‐hypnotic intraoperative dose of propofol of 1 mg/kg infused at 20 mcg/kg/min. Ondansetron was not used as part of routine care except in the blinded fashion as described above.

### 2.3 Postoperative study protocol

A two‐step rescue protocol for symptoms of nausea or pruritus was followed postoperatively (see Appendix A), designed with assistance from anesthesiology pharmacology experts to adhere to the institution's current standard of care for postoperative nausea and vomiting in pediatrics, whilst omitting ondansetron. The first‐line rescue agent for pruritus was nalbuphine (0.03 mg/kg q6H PRN), with second‐line rescue agent of diphenhydramine (0.3 mg/kg q6H PRN). The first‐line rescue agent for PONV was prochlorperazine (0.1 mg/kg q6H PRN), with the second‐line agent of promethazine (3.125 mg <40 kg or 6.25 mg >40 kg q6H PRN). Children who were still symptomatic following these rescue treatments were referred to the on‐call anesthesiologist for assessment; further treatment was at the provider's discretion, with a low‐dose naloxone infusion (dosing range 0.1–3 mcg/kg/min) being recommended. Naloxone bolus was ordered to be given as needed for the treatment of respiratory depression or over‐sedation, per the hospital standard of care for intrathecal morphine.

Trained research assistants, blinded to the intervention, interviewed subjects and sought input from parent or bedside nurse every 6 h for the first 24 h after surgery, with a rest break from 10 pm–7 am. Pruritus was scored as in previous pediatric studies, as 0 = none; 1 = mild/tolerable/not requiring treatment; 2 = severe/intolerable/requiring treatment. Nausea and vomiting was similar scored (0 = no nausea or vomiting; 1 = mild/tolerable nausea/vomiting not requiring treatment; 2 = severe/intolerable nausea/vomiting requiring treatment).^23^ The following data were also collected: all analgesics, anti‐emetic, and anti‐pruritic medications administered. Pain scores were collected at 6‐h intervals, using a 0–10 pain scale, or a rating on the pictorial pain “faces” scale, converted to a 1–10 score.^24^ Patients and parents were also asked an overall pain satisfaction (Yes/No) question as part of institutional protocol. Any recorded adverse events were noted and were additionally reviewed by the authors (EMP and RAH).

A data safety monitoring board planned for an interim analysis at 20 patients per group, to review the incidence of pruritus and nausea in both groups, to ensure pruritus and nausea were adequately treated, and to review adverse events.

### 2.4 Statistical analysis

For analytic purposes, pruritus incidence was dichotomized to be none/mild vs. severe/ treatment required. PONV was similarly categorized. Descriptive statistics were presented as frequencies with percentages for categorical data and either means with standard deviations or medians with 25th and 75th percentiles for continuous data, as appropriate. Data normality was assessed using the Shapiro–Wilks test. Univariate comparisons between the ondansetron and placebo groups were conducted using independent *t*‐tests or Wilcoxon rank‐sum tests for continuous variables, or Chi‐square or Fisher's exact tests for categorical variables, as appropriate. A *p*‐value of < .05 was considered statistically significant for all analyses conducted, and SPSS (version 25, IBM Corp) was used. All analysis was conducted as intention‐to‐treat.

### 2.5 Power analysis

A sample size of 56 patients in each group (total *N* = 112) was deemed necessary to achieve 80% power to determine a reduction in the incidence of pruritus from 40% to 20% (*α* = .05). An assumed incidence of pruritus of 40% in the placebo group was based on a retrospective review of intrathecal morphine side effects in 128 children undergoing these surgeries, at our institution.^2^

## 3 RESULTS

**FIGURE 1:**
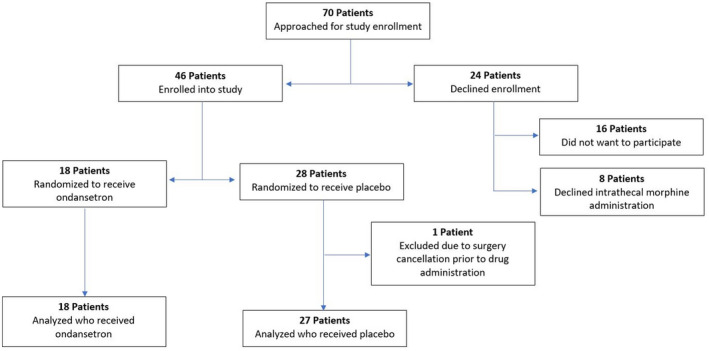
CONSORT flowsheet.

Patients were enrolled from January 2018 to September 2019. Of the 70 patients approached for inclusion in the study, 46 patients enrolled, and were randomized on the day of surgery (Figure 1). At the planned interim analysis, according to the Data Safety Monitoring Plan, the study team was to review results and make decisions regarding any necessary modifications to the study. This review revealed that the incidence of nausea was double in the placebo group, in comparison with the treatment group, mostly requiring treatment. Additionally, it became clear that futility had already been reached because there was such a high rate of pruritus in both the active treatment and placebo groups, with a very small difference in pruritus in the active treatment group. To show that difference to be statistically significant, the study's patient enrollment would need to have been increased in size by a log scale or more, and this was not practical. The study was terminated.

At the time of study termination, 18 subjects were enrolled in the ondansetron group and 28 in the placebo group, one subject in the placebo group was excluded from analysis due to cancellation of surgery after enrollment, but prior to any study drug administration. There was an error in the randomization table that was not discovered until the planned interim analyses at 20 participants per arm was performed. This revealed the unequal randomization. The median age of the cohort was 8.3 years (interquartile range (IQR) 5.8, 9.9) with a median weight of 32.5 kg (IQR 20.4, 38.1) and a majority were female (64%, 29/45). No patients met the high risk PONV criteria outlined in the protocol, no patients received TIVA or preoperative aprepitant. One patient received a sub‐hypnotic intraoperative dose of propofol. There were no significant differences in demographic characteristics of patients in the ondansetron group compared with those in the placebo group (Table 1). Fourteen patients in the ondansetron group (78%) and 22 patients in the placebo group (82%) received urologic surgery. Fourteen patients in the ondansetron group (78%) and 24 in the placebo group (89%) experienced postoperative pruritus (Table 2). There was no statistically significant difference in the risk of any pruritus for the ondansetron group compared with the control group (relative risk (RR) 0.88, 95% CI: 0.66, 1.16; *p* = .412). Those in the ondansetron group had significantly lower risk of postoperative nausea compared with those in the control group (85% vs. 44%, RR 0.52, 95% CI: 0.30, 0.90; *p* = .008). Due to the small sample size, subgroup analysis by gender, age, and surgical subgroups was not possible.

**TABLE 1:** Patient characteristics stratified by study group

	Ondansetron group (*N* = 18)	Saline placebo group (*N* = 27)
Age (years)	8.3 [3.5 to 17.1]	8.2 [3.7 to 16.7]
Weight (kg)	30.4 [16.4 to 64.8]	34.0 [14.5 to 91.4]
Female gender	12 (67)	17 (63)
ASA 1 or 2	15 (83)	27 (100)
Surgical service		
Urology	14 (78)	22 (82)
Orthopedics	4 (22)	5 (19)

*Note*: Data are presented as frequency (percentage) or median [25th percentile to 75th percentile] as appropriate.

**TABLE 2:** Primary and secondary outcomes by study group

	Overall (*N* = 45)	Ondansetron group (*N* = 18)	Saline placebo group (N = 27)	Relative risk or difference between means (95% CI)	*p*‐Value
Pruritus					
Any pruritus	38 (84.4)	14 (77.8)	24 (88.9)	0.88 (0.66, 1.16)	.412
Mild/no treatment required	5 (11.1)	1 (7.1)	4 (16.7)		
Severe/treatment required	33 (73.3)	13 (92.9)	20 (83.3)		
First‐line treatment used	33 (73.3)	13 (92.9)	20 (83.3)		
Second‐line treatment used	11 (28.9)	4 (28.6)	7 (29.2)		
Nausea					
Any nausea	31 (68.9)	8 (44.4)	23 (85.2)	0.52 (0.30, 0.90)	.008
Mild/No treatment required	6 (13.3)	2 (25.0)	4 (17.4)		
Severe/Treatment required	25 (55.6)	6 (75.0)	19 (82.6)		
First‐line treatment used	23 (74.2)	6 (75.0)	17 (73.9)		
Second‐line treatment used	8 (25.8)	1 (12.5)	7 (30.4)		
Pain scores out of 10					
Post Anesthesia Care Unit	1.89 (SD 2.75)	1.78 (SD 2.76)	1.96 (SD 2.79)	−0.18 (−1.88, 1.52)	.858
At 24 h	2.27 (SD 2.15)	2.30 (SD 2.13)	2.25 (SD 2.21)	0.05 (−1.29, 1.39)	.940
Satisfied with pain control? Yes:	44 (97.8)	18 (100%)	26 (96%)	1.04 (0.96, 1.12)	.317

*Note*: Data are presented as frequency (percentage). Percentages for mild/no treatment required and severe/treatment required rows are out of those with the condition of interest. Pain scores are out of 10; presented as Mean and SD.

Pain scores in PACU and at 24 h postoperatively were low, and not significantly different between the ondansetron and placebo groups (Table 2). Across both groups, patient pain satisfaction, or parent proxy report of satisfaction, was high (44/45, 98%).

### 3.1 Protocol deviations and side effects

Of 180 study drugs doses administered, nine doses were delayed (5%), the delay ranging from 30–180 minutes. Two subjects (placebo group) received a “second line” anti‐nausea treatment before or in lieu of “first line” rescue. These results are all included in our intention‐to‐treat analysis.

No severe adverse events were reported, meaning no patients were admitted to intensive care, required naloxone, required oxygen, had respiratory depression, desaturation, over‐sedation or hypotension requiring treatment.

Pruritus was extreme (i.e., not controlled by the rescue medications in the protocol) in two subjects. This resolved with an additional dose of nalbuphine in both subjects. One subject was from each study group. Extreme nausea (i.e., not controlled by the rescue medications in the protocol) occurred in one subject (placebo group); this patient withdrew from study at parent's request, after the second dose of study medication, in order to receive ondansetron. The patient did not meet criteria for preoperative aprepitant, and TIVA or sub‐hypnotic propofol infusion were not used intraoperatively. Nausea in this case did not resolve following IV ondansetron but receded with a second dose of promethazine and time. This subject was included in the intent to treat analysis.

## 4 DISCUSSION

This prospective, randomized, double‐blinded, placebo‐control trial sought to determine if ondansetron would reduce the incidence of pruritus following intraoperative intrathecal morphine administration in children. The study was halted following the interim analysis, as the incidence of pruritus was far higher than expected (84% of all patients) with no difference between the groups, rendering the study futile for determining the primary outcome. Additionally, the incidence of nausea was almost doubled in the saline placebo group, compared with the ondansetron active treatment group (85% vs. 44%). Mainly, the Data Safety Monitoring Plan was put into place to ensure that rates of PONV in the group of patients who were not receiving ondansetron were not higher compared with the patients who were receiving ondansetron. The IRB required this as ondansetron is used in the treatment and prevention of PONV and withholding it from a subset of patients has obvious ethical implications. During this review, it also became clear that futility had been reached because of the unexpectedly high rate of pruritus in both the active treatment and placebo groups. The very small difference in pruritus in the active treatment group was far from meeting criteria for statistical significance given our sample size. Given the small difference in incidence of pruritus between the treatment and placebo groups found, a revised power analysis found the number needed to demonstrate a difference in pruritus between groups as 356 subjects (178/group; with .8 *β*; .05 *α*). Based on rates of patient enrollment thus far, we estimated this would take at least 13 years to complete the study at this single institution, and this was deemed to be unfeasible.

To the best of our knowledge, this is the only randomized control trial in children to prospectively compare a 5‐HT_3_ antagonist to placebo, for the purpose of evaluating pruritus as the primary outcome, following intrathecal morphine. Current literature has found this to be effective in adult populations,^4^, ^5^, ^6^, ^7^, ^8^, ^9^, ^12^, ^19^ but we have been unable to demonstrate this in children; this study suggests a large multi‐center trial would be required to demonstrate any effect. There are limitations of projecting accepted outcomes in adults on to pediatric patients. Research on dose, efficacy, and side effects of many drugs in children is limited, and often extrapolated from adult data. The official statement by the American Academy of Pediatrics on the off‐label use of drugs in children recognizes that this practice is common.^25^ While different models exist for extrapolating data, most studies agree the optimal model for determining pediatric dosing is unclear, and significant limitations exist to directly extrapolating trial results from adult studies.^26^, ^27^

We found the incidence of pruritus to be considerably higher than that reported by previous studies of intrathecal morphine in children, including our own retrospective analysis.^1^, ^2^, ^3^ This finding may be, in part, explained by the prospective nature of the study, with active elucidation of symptoms. Data on the side effects of intrathecal morphine in children, including pruritus and nausea, are typically studied as secondary outcomes (for example, as in our own review: a chart review for use of anti‐pruritics, rather than a symptom scoring scale)^2^, ^3^, ^28^ which may result in underreporting. Additionally, our trial results may have been biased by the frequent assessments of self‐reported outcomes or by the power of suggestion. The term “itch” and the informal title “Anti‐Itch Study” was mentioned numerous times to every subject and their families, and all subjects and their parents and caregivers were repeatedly interviewed about pruritus and nausea symptoms, thus results may have demonstrated bias due to the Hawthorne effect.

Although pruritus was substantially more common than expected, intractable pruritus was not common. All but 2 patients (4.4%) had successful treatment of their pruritus following administration of the rescue agents (1st line nalbuphine, 2nd line diphenhydramine) outlined in our protocol. The choice of anti‐pruritics, other than the omission or inclusion of ondansetron, is common and mirrored our institution's standard of practice. Ondansetron was effective in preventing our secondary outcome, nausea, and vomiting, which was significantly higher in the placebo group, despite routine use of intraoperative anti‐emetics for both groups.

In this study, there were no serious side effects following intrathecal morphine in children, namely no respiratory depression, hypotension, desaturation, intensive care unit admissions, or naloxone administration required. Additionally, we demonstrated good pain relief (based on pain scores and overall pain management satisfaction scores), in agreement with our previous work.^2^ As a result, we suggest that patients and parents should be better informed that treatable pruritus should be expected following intrathecal morphine, but that extreme (hard to treat, or untreatable) pruritus and nausea is uncommon.

This study has several strengths, including the randomized nature of the design within a cohort of individuals who received intrathecal morphine as part of standard of care. This was a prospective study that took place at a single, US academic medical center. Additionally, findings from the study strengthen ondansetron's use as an effective anti‐emetic. Limitations of the study include its early termination, as described above, and the uneven randomization, which occurred due to an error in the randomization table that was not discovered until the planned interim analysis. A large study population would be required to power this study, based on this randomized control trial. Additionally, the subjective nature involved in deciding when to treat pruritus meant the severity of pruritus was not explored, within those who required anti‐pruritic treatment. The risk of interpretive bias of patients and parents' self‐reporting, and interviewer bias in data collection may have influenced the results and could be addressed in future studies by using a constant, blinded observer for quantifiable number of “incidence of scratching,” for example, perhaps studied by age‐based subgroups. Similarly, accuracy of nausea scoring in young children is questionable; often administration of anti‐emetics was used as a surrogate for existence of nausea. Finally, we used our institutional standard dose of 4–5 mcg/kg of intrathecal morphine in this study. While doses this high are not typical in adults, pediatric patients, especially younger ones, will experience a shorter duration of action of the drug due to faster CSF turnover relative to adults. Future studies are needed to determine if decreasing the dose of intrathecal morphine would result in less pruritus while maintaining high levels of analgesia.

In conclusion, this RCT showed that the rates of pruritus following intrathecal morphine administration in our sample were higher than expected. We found no evidence that ondansetron (vs. saline placebo) IV every 6 h prevented this pruritus. However, our secondary outcomes affirmed ondansetron's use in the prevention of postoperative nausea. Physicians, parents, and children should be aware that the unpleasant side effects of pruritus and nausea are common after intrathecal morphine, but these are generally successfully treated.

## AUTHORS' CONTRIBUTIONS

EMP and RAH designed the study, directed the data collection and analysis, and were major contributors in interpreting results and in writing the manuscript. SM assisted with study design and results interpretation. JMP and YL contributed to study design and editing of the manuscript. AL provided statistical analysis and assisted with results interpretation. All authors read and approved the final manuscript.

## FUNDING INFORMATION

This research did not receive any specific grant from funding agencies in the public, commercial, or not‐for‐profit sectors.

## CONFLICTS OF INTEREST

All authors declare no conflicts of interest.

## ETHICS APPROVAL

Registered at ClinicalTrials.gov (NCT03262038); University of Michigan IRB approval HUM00124202 approved 11/30/2017.

## Data Availability

The data that support the findings of this study are available from the corresponding author, EMP, upon request.
